# Assessment of Imatinib Anti-Remodeling Activity on a Human Precision Cut Lung Slices Model

**DOI:** 10.3390/ijms25158186

**Published:** 2024-07-26

**Authors:** Sara Bozzini, Eleonora Bozza, Cecilia Bagnera, Patrizia Morbini, Sara Lettieri, Matteo Della Zoppa, Giulio Melloni, Laura Saracino, Mirko Belliato, Federica Meloni

**Affiliations:** 1 Second Department of Anesthesia and Cardiothoracic ICU[M1] [BS2], IRCCS San Matteo Foundation, 27100 Pavia, Italy; 2 Department of Pediatric Oncoaematology, Cell Factory, IRCCS San Matteo Foundation, 27100 Pavia, Italy; 3 S.C. Anatomia e Istologia Patologica, E.O. Ospedali Galliera, 16128 Genova, Italy; 4 Respiratory Diseases Unit, IRCCS San Matteo Foundation, 27100 Pavia, Italy; 5 Department of Thoracic Surgery, IRCCS San Matteo Foundation, 27100 Pavia, Italy; 6 Department of Cardio-Thoracic, Vascular Sciences and Public Health, University of Padua, 35122 Padua, Italy

**Keywords:** lung fibrosis, precision cut lung slices, imatinib

## Abstract

Recent studies have emphasized the critical role of alteration in cellular plasticity in the development of fibrotic disorders, particularly pulmonary fibrosis, prompting further investigation into molecular mechanisms and therapeutic approaches. In this context, Precision Cut Lung Slices (PCLSs) emerge as a valuable ex vivo research tool. The process of PCLSs generation preserves most features of the naïve lung tissue, such as its architecture and complex cellular composition. We previously stimulated normal lung PCLSs with two different stimuli (fibrotic cocktail, composed by platelet lysate and TGFβ, or neutrophil extracellular traps) and we observed a significant elevation of Epithelial–Mesenchymal Transition (EMT) markers from 24 h to 72 h of culture. The aim of our work was to exploit this PCLSs based ex vivo model of EMT, to evaluate the effect of imatinib, an old tyrosine kinase inhibitor with reported anti-remodeling activities in vitro and in animal models. Imatinib treatment significantly decreased α-SMA and collagen expression already starting from 24 h on stimulated PCLS. Imatinib showed a significant toxicity on unstimulated cells (3-fold increase in *ACTA2* expression levels at 24 h, 1.5-fold increase in *COL1A1* expression levels at 24 h, 2-fold increase in *COL3A1* expression levels at 72 h). Further evaluations on specific cell lines pointed out that drug effects were mainly directed towards A549 and LFs. In conclusion, our model confirms the anti-remodeling activity of imatinib but suggests that its direct delivery to alveolar epithelial cells as recently attempted by inhalatory preparation of the drug might be associated with a non-negligible epithelial cell toxicity.

## 1. Introduction

Interstitial lung fibrosis includes a group of fibrogenic lung disorders characterized by progressive scarring of the lung parenchyma, with remodeling and architectural distortion, impaired gas exchange and, eventually, death. The prognosis of the disease is inauspicious, with a median survival of 3–5 years. Antifibrotic drugs available for the treatment slow disease progression but do not reverse fibrotic damage. This emphasizes the need of new generation of such drugs [1]. The conventional pathogenesis of the disease results from repeated injuries to airway epithelial cells (due to exposure to inhalatory noxious agents, drugs or immune-inflammatory insults) triggering the process of epithelial transition towards myofibroblasts. This ends in a fibro-proliferative phase with uncontrolled deposition of extracellular matrix [2].

Evidence of epithelial plasticity ranges from metaplasia and transdifferentiation in the major airways with viral infections to hyperplasia and potentially Epithelial–Mesenchymal Transition (EMT) in the lung parenchyma during fibrogenesis [3,4,5]. The molecular mechanisms underlying EMT and interstitial collagen deposition are only partially clarified. In the context of required investigation of molecular mechanisms in pulmonary fibrosis, Precise Cut Lung Slices (PCLSs) constitute a useful tool. They reproduce the tridimensional structure of the native lung and maintain cell-to-cell and cell-to-matrix interactions in the context of the complex architecture of the lung. Thus, recently, they have gained attention as new models of human disease appropriate for evaluating treatment efficacy and studying molecular mechanisms in a whole-tissue model [6]. We recently developed and utilized PCLSs obtained from tumor-free tissue of non-smokers patients without IPF diagnoses to establish an ex vivo model of cellular plasticity modulation [7]. In this model, plasticity alteration is induced by the use of either a fibrotic cocktail (FC) composed of Platelet Lysate (PL) and TGFβ, or NETs (Neutrophil Extracellular Traps).

The evaluation of novel therapies for Idiopathic Pulmonary Fibrosis (IPF) treatment using PCLSs is currently underway [8]. We aimed to assess, using this model, imatinib mesylate, a potent and specific tyrosine kinase inhibitor, developed for the treatment of chronic myeloid leukemia and gastrointestinal stromal tumors [9]. Several data in the literature have demonstrated that imatinib has the potential to prevent pulmonary fibrosis by inhibiting the proliferation of mesenchymal cells, and that it might be a useful anti-remodeling agent in several fibrogenic contexts, including human lung fibrosis [10,11,12,13,14].

## 2. Results

### 2.1. Ex Vivo Lung Fibrosis Model: Histopathological Validation

**Figure 1:**
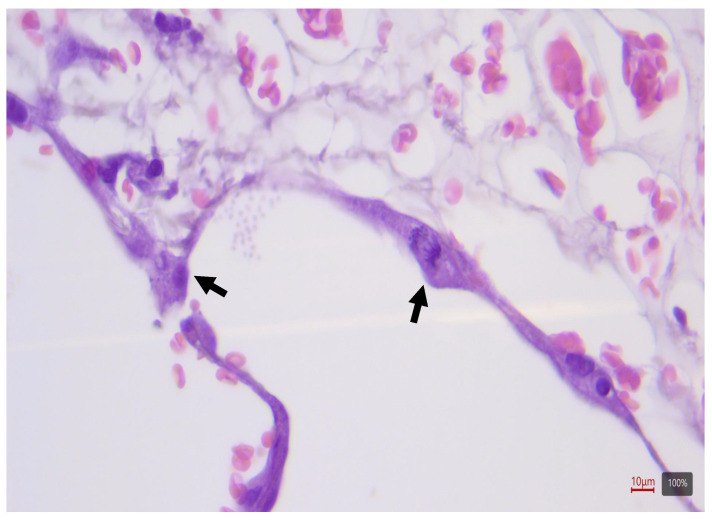
Representative image of 3-micron-thick paraffin sections stained with hematoxylin and eosin after 72 h of culture with activated type 2 pneumocytes and mitoses indicated by black arrows. Scale bar 10 μm, magnificence 80×.

**Figure 2:**
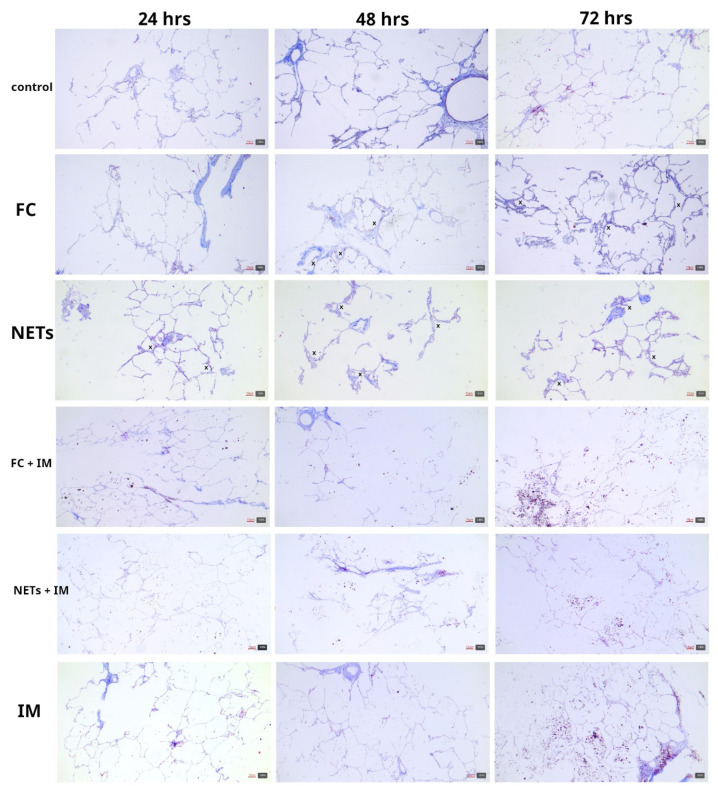
Representative microphotographs showing lung PCLSs treated with different stimuli and imatinib at different time points. PCLS incubation, FC, NETs, or IM treatment did not affect cell and tissue viability. A slight increase in interstitial connective tissue and cellularity was observed with both treatments at 24 to 72 h (thickened alveolar septa highlighted with X mark), while IM treatment appeared to prevent thickening of alveolar septa. IM alone did not affect lung architecture (Masson trichrome stain, 4× magnification, scale bar 10 μm).

We previously assessed the PCLS model from lung resection segments by vibratome cutting to obtain slices of 300–350 μm thickness. To induce fibrosis, PCLSs were cultured for 24, 48, and 72 h with FC or NETs able to induce alterations in cellular plasticity in PCLSs, documented by increased α-SMA (*ACTA2*) and collagen deposition, as determined by the upregulation of type 1a1 collagen (*COL1A1*) and type 3a1 collagen (*COL3A1*) tissue levels. FC or NET treatment did not significantly affect tissue viability at all time points and the upregulation of these fibrotic markers demonstrated that the ex vivo lung EMT model was successfully established [7]. In the present work, we have confirmed from a histopathological point of view the previous findings of increased expression levels by real-time PCR of the investigated EMT markers. Histological analyses showed that cultured PCLSs maintained intact lung architecture up to 72 h, where activated type 2 pneumocytes and mitoses were observed (Figure 1). FC and NET treatment did not affect cell and tissue viability, even if a slight increase in interstitial connective tissue and cellularity was observed with both treatments at 24 to 72 h (Figure 2).

### 2.2. Imatinib Modulation of Induced Fibrosis in Lung PCLS

#### 2.2.1. Determine Optimal Imatinib Concentration and IC50 Value

In order to determine the concentration of imatinib useful for induced-PCLS treatment, we determined the IC50 value of imatinib in LFs by treating them with different concentrations of imatinib for 72 h and then performing an MTT assay (Supplementary Figure S1). The evaluation was conducted on LFs, as their aberrant proliferation have been proven to play a pivotal role in Interstitial Lung Disease (ILD) pathogenesis and progression together with the profibrotic signals mediated by TGFβ [15]. In addition, these are primary cells from ILD patients isolated from Bronchoalveolar Lavage and thus representative of the actual target of imatinib treatment.

#### 2.2.2. EMT Marker mRNA Levels

**Figure 3:**
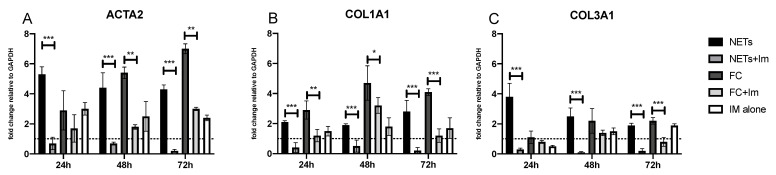
Quantitative analyses of *ACTA2* (**A**), *COL1A1* (**B**) and *COL3A1* (**C**) in PCLSs of ex vivo model using NETs or FC as stimuli and then treated with imatinib. The results are presented as the mean ± SD of the fold change relative to GAPDH from the lung cancer resection tissue of ten patients, incubated for 24, 48, or 72 h. The data are referenced to untreated control PCLSs, represented in the figure as the dashed line. *** *p* < 0.0001; ** *p* < 0.001; * *p* < 0.01.

Following the above-summarized results, we designed an experimental setting to verify if imatinib treatment is able to modulate these effects ex vivo. Treatment with 15 μM imatinib induced a marked reduction in NET-induced *ACTA2* mRNA levels at each evaluated time (Figure 3A) and *COL1A1* and *COL3A1* mRNA levels were largely reduced by imatinib in NETs-induced PCLSs (Figure 3B,C). In PCLSs treated with FC, a decrease in *ACTA2* mRNA was observed from 48 h to 72 h in the presence of imatinib, even if it did not return to baseline levels (Figure 3A). *COL1A1* mRNA levels were reduced at 24–72 h in PCLSs treated with FC (Figure 3B), while *COL3A1* mRNA levels were significantly influenced by the presence of imatinib only at 72 h (Figure 3C).

Quite unexpectedly, control PCLSs treated with imatinib without any stimulation showed a significant increase in *ACTA2* mRNA levels and a slight increase in *COL1A1* and *COL3A1* mRNA levels (Figure 3).

#### 2.2.3. Cytokine Release

**Figure 4:**
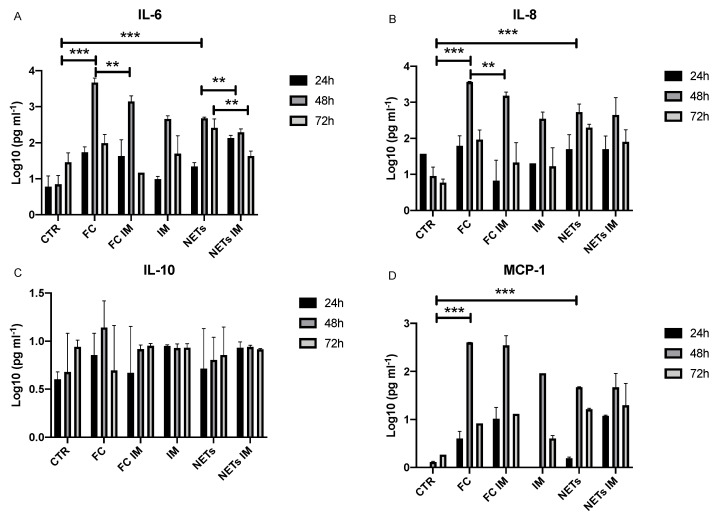
Cytokine release by PCLS. IL-6 (**A**), IL-8 (**B**), IL-10 (**C**) and MCP-1 (**D**) levels in the supernatant of PCLS cultures. Cytokine concentrations are expressed as Log10 (pg mL^−1^). The results are shown as mean  ±  SD of values from three determinations. *** *p* < 0.0001; ** *p* < 0.001.

In the supernatant of the PCLS cultures, we evaluated the release of some pro-inflammatory/fibrogenic cytokines: IL-6, IL-8, IL-10, and MCP-1. After 48 h of treatment, both FC and NET stimulation induced a marked increase in IL-6, IL-8, and MCP-1 levels (Figure 4). Levels of different growth factors (such as TGF, FGF, VEGF, etc.) were not determined because many of these are contained in the platelet lysate used to stimulate slices in FC, and levels could be affected by their presence in the culture medium.

The addition of imatinib induced a significant reduction in IL-6 levels after 48 h in both stimulation models (Figure 4A) and after 72 h for NTEs-PCLS. Similar results were observed regarding the release of IL-8 for PCLSs treated with FC but not with NET stimulation (Figure 4B). No significant increase in IL-10 was observed with respect to baseline (Figure 4C). Imatinib does not appear to affect MCP-1 release (Figure 4D).

### 2.3. Imatinib-Induced Cytotoxicity on PCLSs and Lung Cells

**Figure 5:**
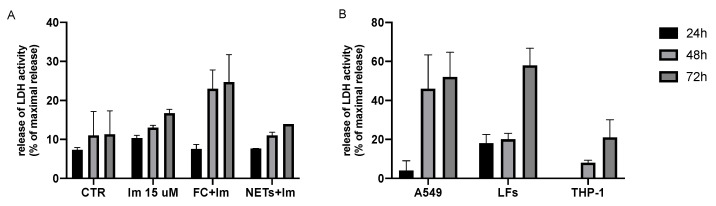
LDH assay results in PCLSs (**A**) and cells (**B**). LDH release was detected using CytoTox 96^®^ Non-Radioactive Cytotoxicity Assay. All experiments were conducted in triplicate. The experimental data are presented as % maximal release of LDH.

PCLS supernatants were collected from 24 h to 72 h in order to better understand the mechanisms underlying the increase in *ACTA2*, *COL1A1* and *COL3A1* expression levels in unstimulated PCLSs treated with imatinib at 15 μM. Metabolic assessment was performed by determining LDH release and it was confirmed that no major cell death occurred up to 72 h (Figure 5A). Imatinib, even with the combination of a stimulus, whether NETs or FC, did not induce an increase in cytotoxicity. While a greater increase in release was observed in the presence of FC + Im at 48 h and 72 h, it did not exceed 25% of maximal release.

Histopathological examination of PCLSs exposed to imatinib alone showed that they qualitatively retained their structure and did not show an increase in interstitial collagen compared to untreated controls (Figure 2).

We decided to perform cytotoxicity tests also on homogeneous cultures of the most representative cell types present in lung tissue using a commercial type II pneumocyte cell line (A549), macrophages derived from the THP-1 cell line, and primary Lung Fibroblasts (LFs) obtained in our laboratory from broncholaveolar lavage of fibrotic patients. Imatinib at 15 μM concentration showed different cytotoxicity in different cell types (Figure 5B). In particular, in A549 cells, cytotoxicity reached its plateau at 48 h (46% of maximal release), which was maintained up to 72 h (52% of maximal release). The same effect was detectable on LFs and THP-1, where the cytotoxicity peak was delayed at 72 h (58% and 21% of maximal release, respectively).

### 2.4. EMT Marker Expression after Imatinib Treatment of Lung Cells

**Figure 6:**
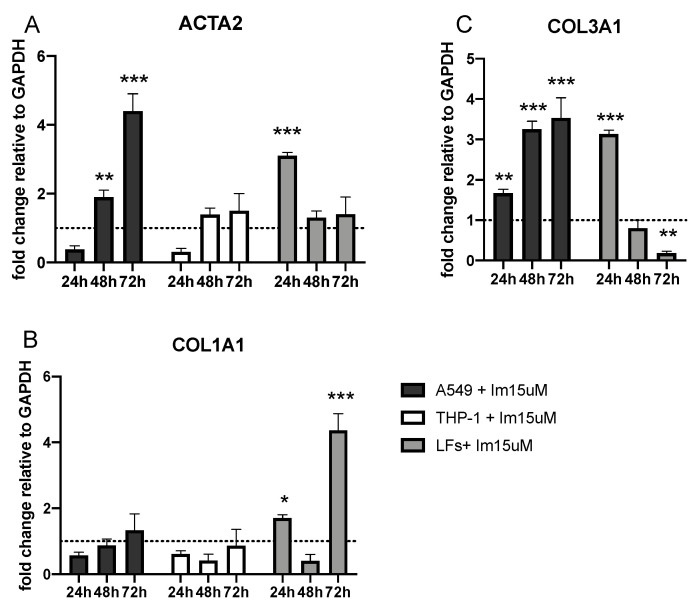
Quantitative analyses of *ACTA2* (**A**), *COL1A1* (**B**) and *COL3A1* (**C**) in A549, THP-1, and LFs treated with imatinib. The results are shown as mean ±  SD of the fold change relative to GAPDH from three independent experiments after 24–48–72 h of incubation. The data are referenced to untreated control, represented in the figure as the dashed line. *** *p* < 0.0001; ** *p* < 0.001; * *p* < 0.01.

Treatment with imatinib 15 μM was shown to induce a significant increase in αSMA mRNA expression levels in A459 cells after 48 h with respect to untreated controls (1.9-fold change), with a further increase after 72 h of treatment (4.4-fold change). In LFs, αSMA mRNA overexpression was observed at 24 h (3.1-fold change), while in THP-1 no significant difference was observed between treated and untreated cells (Figure 6A).

*COL1A1* mRNA expression was significantly upregulated in LFs at 24 h (1.7-fold change) and further increased at 72 h (4.4-fold-change) (Figure 6B). In A549 and THP-1, no significant differences were observed between treated and untreated cells with respect to *COL1A1* expression levels.

In A549, *COL3A1* mRNA levels increased significantly after 24 h up to 72 h (1.7-fold change and 3.5-fold change, respectively). *COL3A1* overexpression was observed in LFs after 24 h of treatment with imatinib (3.1-fold change), and it returned to levels similar to those of untreated cells after 48 h. THP-1 showed no expression of *COL3A1* (Figure 6C).

## 3. Discussion

Interstitial lung fibrosis might be idiopathic or secondary to inflammatory/auto-alloimmune insults. This disease is characterized by progressive and irreversible replacement of lung parenchyma by scar tissue leading to respiratory failure and early mortality. The idiopathic form is considered a pathology of senescence, and with the extensive aging of the population, it is expected to impose major health and financial burdens on patients and society in the near future [16].

The complex etiology and cellular pathomechanisms of this disease remain poorly understood [1,17] and the anti-fibrotic drugs currently available for the treatment of IPF have been demonstrated to slow disease progression but do not reverse fibrotic damage [18,19]. Moreover, they are burdened by relevant extrapulmonary side effects, since they are administered by the systemic route. This emphasizes the need to develop new drugs, which is difficult due to the lack of animal models that can reproduce the complexity of human populations [20]. On another hand, human trials seldom have failed due to the difficulties of tested molecules to capture heterogeneity and pleiotropic pathways of IPF. To overcome this limit, models using human lung tissue have been developed [6,7,8]. PCLSs provide an emerging and exciting opportunity to advance research in the field of pulmonary fibrosis and test novel therapies on whole lung tissue [21]. Tissues obtained from explanted lungs with established fibrosis represent a late stage of the disease process. Consequently, models of disease generated on normal tissue might offer better chances for drug screening. PCLSs from normal lungs exposed to TGF-β and various profibrotic stimuli demonstrated relevant changes comparable to those observed in the early remodeling stages. These included the upregulation of pro-fibrotic genes, an increased thickness of alveolar septa, and aberrant activation of pulmonary cells [6,22]. Our model analogously reproduces early EMT stages of lung fibrogenesis with two simple and innovative stimulation strategies [7]. The model is associated with the expression and deposition of collagen, as evidenced by histological examination of stimulated PCLS. Additionally, the results of the histopathological examination corroborate those of the metabolic analysis, indicating the absence of significant necrosis.

PCLSs have been adopted for pre-clinical drug discovery and toxicological studies [23,24]. In this study, we decided to assess in PCLSs the imatinib effect, a potent anti-fibrotic drug leading to a reduction in cell proliferation, anti-apoptotic signaling, and migration [9,11]. Imatinib’s antifibrotic effect has already been demonstrated on the bleomycin-induced pulmonary fibrosis murine model [10], and recently, it has been reported to stabilize lung function in children with Bronchiolitis Obliterans Syndrome after allogeneic bone marrow transplantation [11,12,13,14]. In addition, this drug is under evaluation in other severe lung diseases such as pulmonary hypertension and severe asthma and its local delivery by inhalatory route has been developed [25]. In our study, imatinib significantly reduced αSMA and collagen expression in PCLSs treated with both FC and NETs, confirming its significant anti-remodeling activity. The rationale for utilizing a fibrotic cocktail, comprising growth factors, inflammatory cytokines, and signaling molecules, was based on its established role in pathohistological alterations that bear resemblance to early lung fibrosis. These alterations include the upregulation of pro-fibrotic genes, thickening of alveolar septa, and aberrant activation of pulmonary cells [26]. In contrast, NET-related injuries were typically investigated within the context of the endothelium [27,28]. The capability of NETs to drive the Endothelial–Mesenchymal Transition has been subjected to comprehensive analysis [29]. However, recent reports have indicated that the infiltration of neutrophils in the alveolar space may interfere with cell–cell adhesion of lung epithelial cells [30]. Additionally, NETosis has been demonstrated to induce EMT efficiently [2]. In the literature, numerous other stimuli have been applied to the PCLS model, including oxidants [31] and cigarette smoke extract [32]. However, in our model, we sought to focus on the well-described TGF-induced mechanism and to confirm the role of NETs in tissue.

Imatinib shows a potent anti-inflammatory effect in our model. Since persistent inflammation results in proinflammatory proteases, chemokines, and cytokines that foster ongoing fibrosis [33,34], the observation that imatinib is able to down-modulate the release of IL-6 and IL-8 after FC stimulation and that of IL-6 alone after NET stimulation provides evidence of another important therapeutic mechanism.

Nevertheless, the administration of imatinib to unstimulated PCLSs resulted in a notable elevation in αSMA and collagen mRNA expression, which became evident as early as 24 h into the culture period. As recently reported, numerous drugs (including anticancer ones, such as methotrexate or geftinib, but also antiarrhythmics such as amiodarone) exert their toxicity on lung epithelial cells by inducing cell cycle arrest and increasing the expression of αSMA in A549 cells) [35]. Whatever the mechanism by which imatinib exerts its toxicity on lung cells, it does not seem to induce a significant degree of collagen deposition, since the histological evaluation of imatinib-treated PCLSs did not detect an increase in collagen deposition at 72 h. This could be due to a temporal mismatch between gene expression and actual protein deposition that can be observed by histopathological examination.

Thus, in the hypothesis that Imatinib could exert its toxicity on epithelial cells we analyzed different cell lines. All tested cell subtypes showed a consistent degree of cytotoxicity at 72 h and the increase in αSMA and collagen expression was detectable in both A549 and in LFs. A limitation of present study was the lack of an evaluation of imatinib effect on lung endothelial cells. This is due to the unavailability of an immortalized lung endothelial cell line. However, it seems likely that imatinib toxicity might involve also lung endothelial cells as has been reported in animal model of lung injury [36]. In addition, it has been recently reported that another tyrosine kinase inhibitor, Dasatinib, is able to exert a significant lung endothelial toxicity [37]. In synthesis, our data suggest that imatinib causes cytotoxicity and prompts the expression of αSMA and collagen in alveolar epithelial cells, thus suggesting that the direct administration of the drug by inhalation route might be associated with some degree of lung injury with long-term administration. Therefore, we can infer that by developing nano-carriers of this drug, we could target specific pathogenic cells and spare normal lung epithelial or endothelial cells from direct toxicity.

## 4. Materials and Methods

### 4.1. Lung Specimens

The use of human tissue was approved by the IRCCS San Matteo Hospital Foundation ethic committee. All patients gave informed consent to the study participation. Macroscopically tumor-free tissue from lung cancer resection surgeries of ten patients with negative imaging for IPF/ILD was used. The histotypes of the lung cancers included adenocarcinoma, squamous cell carcinoma, and undifferentiated cell carcinoma. The specimens were selected from portions distal to the neoplasms, and the absence of neoplastic cells and other significant alterations (i.e., inflammation, granulomas, scarring) was confirmed at histopathological examination.

### 4.2. Lung Slice Preparation and Culture

PCLSs were prepared in accordance with a previously described method [7]. Briefly, the lung resection segment was inflated with warm buffer-equilibrated agarose solution through bronchioles using an appropriate volume according to lung size. After infusing agarose into the lung, cylindrical cores of the lung were prepared with specific tissue coring tools and refrigerated, followed by vibratome cutting (COMPRESSTOME^®^ VF-210-0Z, Precisionary, Ashland, MA, USA) to obtain slices of 300–350 μm thickness.

The PCLSs were incubated in high glucose Dulbecco’s modified Eagle medium (DMEM, Gibco, Life Technologies, Milan, Italy), with 10% fetal bovine serum (FBS, Euroclone, Milan, Italy), 100 U/mL penicillin/streptomycin (P/S) solution, and 100 U/mL L-glutamine (Corning Costar, Glendale, AZ, USA) in multi-well plates at 37 °C, 5% CO_2_ and 95–100% air humidity under tissue culture conditions; the medium was changed daily.

To induce fibrosis, the PCLSs were cultured for 24, 48, and 72 h with either a fibrogenic cocktail containing 3% platelet lysate and 10 ng/mL TGFβ in DMEM supplemented with 1% FBS, or total medium and purified Neutrophil Extracellular Traps isolated following a previously described protocol [2]. Briefly, to induce NETosis, 2.5 × 10^6^ neutrophils were cultivated with the addition of 100 nM PMA (Sigma-Aldrich, St. Louis, MO, USA) for 4 h at 37 °C.

### 4.3. Cell Cultures

A549 epithelial cells (purchased from ATCC^®^, Manassas, VA, USA) were cultivated in high-glucose DMEM supplemented with 10% FCS, 100 U mL^−1^ P/S solution, and 100 U mL^−1^ L-glutamine.

Lung Fibroblasts (LFs) were isolated from Bronchoalveolar Lavage fluids of patients affected by ILD. Briefly, 6 × 10^6^ cells were seeded in the same culture medium and single foci formed between 1–3 weeks were isolated and cultivated [38].

A human monocytic leukemia cell line (THP-1 cells, purchased by ATCC^®^, Manassas, VA, USA) was maintained in RPMI medium supplemented with 10% FCS and 100 U mL^−1^ P/S solution. To induce macrophage differentiation, the THP-1 cells were stimulated with PMA for 48 h.

### 4.4. MTT Assay for Cytotoxicity

LFs were cultured with imatinib in different concentrations (1–500 μM). After 24–48–72 h of treatment, cells were incubated with MTT (3-(4,5-dimethylthiazol-2-yl)-2,5-diphenyltetrazolium bromide, (Sigma-Aldrich—St. Louis, MO, USA) solution media for 4 h. At the end of incubation, 100 μL of DMSO was added to each well. The absorbance was measured in a microplate reader at a wavelength of 550 nm with background subtraction at 630 nm. All experiments were run in triplicate and cell viability was calculated as the percentage of viable control cells. IC50 values were estimated from results of the MTT test described as the drug concentrations that reduced absorbance to 50% of control values.

### 4.5. PCLSs and Cell Imatinib Treatment

After induction with FC or NETs for 24 h, some of the PCLSs were treated with 15 µM imatinib for 24, 48, and 72 h, then supernatants were collected and the PCLSs washed with PBS and harvested for RNA isolation or histological analyses. As a control, a series of PCLSs was treated with imatinib without previous induction of fibrosis.

Cells were seeded into 6-well plates at a density of 1 × 10^5^ cells/well and treated with 15 µM of imatinib for 24–48–72 h.

### 4.6. Cytotoxicity Assays

PCLSs and cell supernatants were collected at 24 h to 72 h and metabolic assessment was performed. After centrifugation at 400× *g* at 4 °C for 5 min, LDH activity was detected using CytoTox 96^®^ Non-Radioactive Cytotoxicity Assay (Promega, Madison, WI, USA) according to the manufacturer’s protocol. During the experiment, three wells were used per experimental condition. Semi-quantitative evaluation of alveolar septa modifications was performed on Masson-trichrome-stained slides by an experienced lung pathologist (PM). The characteristics of tissue and stain did not allow for quantitative evaluations.

### 4.7. Histopathological Evaluation

Untreated control PCLS, PCLSs induced with FC and with NETs, PCLSs treated with imatinib, and PCLSs treated with Imatinib after induction of fibrosis with FC and with NETs were collected for histopathological evaluation at 24, 48, and 72 h. PCLSs for histopathological evaluation were fixed for 24 h with the instillation of 10% buffered formalin in the incubation wells. The slices were then carefully removed, labeled and processed following routine procedures. After paraffin embedding, taking care of maintaining the PCLSs flat at the bottom of the mold, 3-micron-thick paraffin sections were cut by a trained technician, collected onto positively-charged slides and stained with hematoxylin and eosin and Masson trichrome. 

### 4.8. Quantitative RT-qPCR

Total RNA was isolated from cells using an RNeasy Mini Kit (Qiagen, Hilden, Germany) and from PCLSs using miRNeasy Tissue/Cells Advanced Kits (Qiagen, Hilden, Germany). RNA concentration and purity were evaluated using a spectrophotometer (Nanodrop 2000, Thermo Scientific, Madison, WI, USA). cDNA was retro-transcribed from 1 µg of total RNA using LunaScript RT SuperMix Kit (NEB), according to the manufacturer’s instructions. Relative levels of Alpha Smooth Muscle Actin (*ACTA2*), type 1a1 collagen (*COL1A1*), and type 3a1 collagen (*COL3A1*) mRNA were assessed using SYBR^®^ Green Luna^®^ Universal qPCR Master Mix (NEB) and normalized to the levels of glyceraldehyde-3-phosphate dehydrogenase (*GAPDH*) mRNA. All reactions were performed on an LC480 Real-Time PCR system (Roche Diagnostics, Vienna, Austria) according to the manufacturer’s recommendations. Each experiment was performed in triplicate. The threshold cycle (Ct) was defined as the fraction cycle number at which fluorescence exceeded the given threshold. Relative gene expression level quantification was compared with internal standards and analyzed using the 2^−∆∆Ct^ method.

### 4.9. ELISA Assays

IL-8, IL-6, IL-10, and MCP-1 were quantified with a SimpleStep ELISA^®^ Kit (Abcam, Cambridge, UK) following the manufacturer’s instructions; the results are expressed as pg ml-1. Briefly, 50 μL of each sample was added to ELISA kit wells with the addition of 50 μL of antibody cocktail. After incubation at room temperature on a plate shaker, the plates were washed three times to eliminate the unbound antibody. The substrate (100 μL) was incubated for 10 min in the dark at room temperature on a plate shaker, followed by 100 μL of stop solution to read the absorbance at 450 nm.

### 4.10. Statistical Analysis

The mean and standard deviation or median and interquartile range were presented for continuous variables, and numbers and percentages were presented for categorical variables. Groups were compared to parametric or non-parametric tests, according to data distribution, for continuous variables. Statistical analyses were performed using GraphPad Prism version 8 (GraphPad Software, Inc., San Diego, CA, USA). All statistical tests were two-sided, and a *p*-value < 0.05 was considered statistically significant.

## 5. Conclusions

We developed and optimized two ex vivo models of induced fibrosis in lung PCLSs and demonstrated the anti-fibrotic activity of imatinib in this system. Treatment of fibrotic PCLSs with imatinib seems to reduce α-SMA and collagen production, although it shows cytotoxicity. The anti-fibrotic activity of imatininb is possibly mediated by its role in the modulation of inflammatory cytokine release. The direct administration of the drug by the inhalatory route may be associated with significant lung toxicity with long-term administration. We can hypothesize that, by developing targeted carriers of the drug, we could direct the treatment specifically to those cells, which are considered pathogenic in a specific disease context, sparing normal lung.

Altogether, our results show that the induced lung PCLS fibrosis model may represent a valuable system for evaluate potential novel anti-fibrotic drugs for the treatment of pulmonary fibrosis.

## Data Availability

The original contributions presented in the study are included in the article/Supplementary Material, further inquiries can be directed to the corresponding author.

## Supplementary Materials

The following supporting information can be downloaded at: https://www.mdpi.com/article/10.3390/ijms25158186/s1.
