# Supplementary material for: Assessment of Imatinib Anti-Remodeling Activity on a Human Precision Cut Lung Slices Model
Source: Int J Mol Sci. 2024 Jul 26;25(15):8186. doi: 10.3390/ijms25158186 (PMC11311718; doi:10.3390/ijms25158186)

International Journal of
Molecular Sciences

MDPI

Article

Assessment of Imatinib anti-remodeling activity on a human Precision cut lung slices model

Sara Bozzini[1,] *, Eleonora Bozza[2], Cecilia Bagnera[2], Patrizia Morbini[3], Sara Lettieri[4], Matteo Della Zoppa[4], Giulio Melloni[5], Laura Saracino[4], Mirko Belliato[1] and Federica Meloni[6]

[1]Second department of Anesthesia and Cardiothoracic ICU, IRCCS San Matteo Foundation, Pavia, Italy.
[2] Department of Pediatric Oncoaematology/Cell Factory, IRCCS San Matteo Foundation, Pavia, Italy.
[3] S.C. Anatomia e Istologia Patologica, E.O.Ospedali Galliera, Genova, Italy
[4] Respiratory diseases Unit, IRCCS San Matteo Foundation, Pavia, Italy.
[5] Department of Thoracic Surgery, IRCCS San Matteo Foundation, Pavia, Italy
[6] Department of Cardio-Thoracic, Vascular Sciences and Public Health, University of Padua, Padua, Italy.
* Correspondence: s.bozzini@smatteo.pv.it

Supplementary figure S1: IC50 value of imatinib in LFs by treating them with different concentrations of imatinib for 72h and then performing MTT assay. Data showed IC50 Imatinib 24h: 34.53 µM; IC50 Imatinib 48h: 29.48 µM; IC50 Imatinib 72h: 25.65 µM.

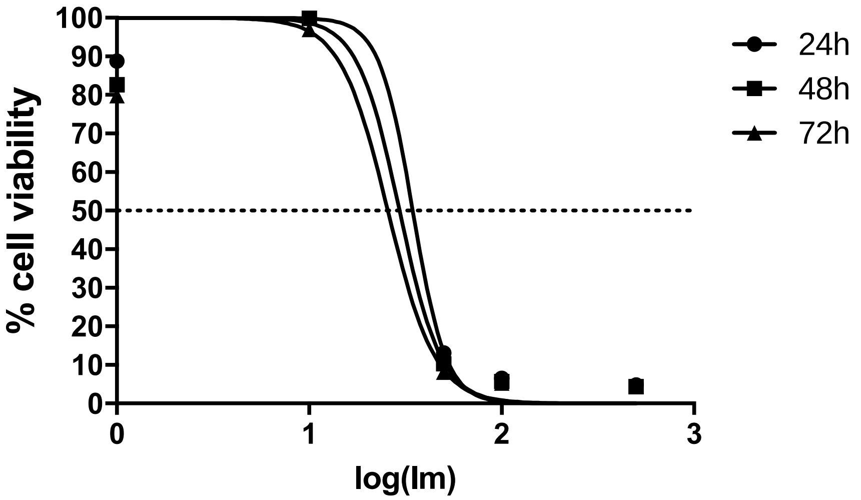

Supplement: Supplementary file 1 [file ijms-25-08186-s001.zip › ijms-3104522-supplementary.pdf]
